# Coherent diffraction microscopy at SPring-8: instrumentation, data acquisition and data analysis

**DOI:** 10.1107/S0909049510051733

**Published:** 2011-01-21

**Authors:** Rui Xu, Sara Salha, Kevin S. Raines, Huaidong Jiang, Chien-Chun Chen, Yukio Takahashi, Yoshiki Kohmura, Yoshinori Nishino, Changyong Song, Tetsuya Ishikawa, Jianwei Miao

**Affiliations:** a Department of Physics and Astronomy and California NanoSystems Institute, University of California, Los Angeles, CA 90095, USA; b RIKEN SPring-8 Center, 1-1-1 Kouto, Sayo, Hyogo 679-5148, Japan; c State Key Laboratory of Crystal Materials, Shandong University, Jinan 250100, People’s Republic of China; d Frontier Research Base for Global Young Researchers, Frontier Research Center, Graduate School of Engineering, Osaka University, 2-1 Yamada-oka, Suita, Osaka 565-0871, Japan; e Research Institute for Electronic Science, Hokkaido University, Kita 21 Nishi 10, Kita-ku, Sapporo 001-0021, Japan

**Keywords:** coherent diffraction microscopy, coherent diffraction imaging, lensless imaging, oversampling, phase retrieval

## Abstract

Since the first demonstration of coherent diffraction microscopy in 1999, this lensless imaging technique has been experimentally refined by continued developments. Here, instrumentation and experimental procedures for measuring oversampled diffraction patterns from non-crystalline specimens using an undulator beamline (BL29XUL) at SPring-8 are presented. In addition, detailed post-experimental data analysis is provided that yields high-quality image reconstructions. As the acquisition of high-quality diffraction patterns is at least as important as the phase-retrieval procedure to guarantee successful image reconstructions, this work will be of interest for those who want to apply this imaging technique to materials science and biological samples.

## Abstract 2

An instrumentation and data analysis review of coherent diffraction microscopy at SPring-8 is given. This work will be of interest to those who want to apply coherent diffraction imaging to studies of materials science and biological samples.

## 1. Introduction

Coherent X-ray diffraction microscopy (CXDM) is a rapidly advancing imaging technique whereby aberration-free diffraction-limited images can be obtained without using lenses (Miao *et al.*, 1999 ▶, 2002 ▶, 2003 ▶, 2006 ▶; Robinson *et al.*, 2001 ▶; Spence *et al.*, 2002 ▶; Williams *et al.*, 2003 ▶, 2006 ▶; Marchesini *et al.*, 2003 ▶; Nugent *et al.*, 2003 ▶; Shapiro *et al.*, 2005 ▶; Pfeifer *et al.*, 2006 ▶; Chapman *et al.*, 2006*a* ▶,*b* ▶; Rodenburg *et al.*, 2007 ▶; Sandberg *et al.*, 2007 ▶, 2008 ▶; Abbey *et al.*, 2008 ▶; Jiang *et al.*, 2008 ▶, 2010 ▶; Thibault *et al.*, 2008 ▶; Song *et al.*, 2008 ▶; Barty *et al.* 2008 ▶; Nishino *et al.*, 2009 ▶; Huang *et al.*, 2009 ▶; Lima *et al.*, 2009 ▶; Ravasio *et al.*, 2009 ▶; Mancuso *et al.*, 2009 ▶; Raines *et al.*, 2010 ▶; Newton *et al.*, 2010 ▶; Takahashi *et al.*, 2010 ▶; Nelson *et al.*, 2010 ▶; Giewekemeyer *et al.*, 2010 ▶). Such images are computed directly from the diffracted intensities measured in the far-field. Under proper illumination and experimental configuration, these measurements are proportional to the square modulus of the sample’s Fourier transform and are referred to as the diffraction pattern of the sample. Recovering the image requires solving for the phases associated with the Fourier coefficients. A solution exists if the measurements are sampled at a frequency finer than the inverse of the sample size (*i.e.* oversampled). When the diffraction pattern is oversampled such that the number of independently measured intensity points exceeds the number of unknown variables of the sample density, the phases are in principle encoded in the diffraction intensity (except for some special or trivial cases) (Miao *et al.*, 1998 ▶) and can be retrieved by iterative algorithms in combination with general physical constraints such as finite extent and non-negativity (Fienup, 1982 ▶; Elser, 2003 ▶; Chen *et al.*, 2007 ▶; Marchesini, 2007 ▶).

This imaging technique has several advantages related to a probing field as well as a probed sample. X-rays are difficult to focus, and optics-based imaging systems that rely upon such focusing components only achieve resolutions limited by their fabrication precision, which are currently limited to around 10 nm (Mimura *et al.*, 2010 ▶). However, by using CXDM there is no such resolution limitation and, in principle, diffraction-limited images may be obtained as a function of incident wavelength and detector field of view, fulfilling the first-order Born approximation. Additionally, whereas electron-based microscopes can obtain high-resolution images, they are also limited by stringent constraints upon sample thickness and the multiple-scattering effect. Owing to the relatively longer penetration depth of hard X-rays, CXDM can be used to non-invasively probe the three-dimensional structures of samples that are a few micrometres thick. To date, a broad range of samples have been studied by CXDM, including those of nanocrystals, porous materials, yeast cells, bacteria, human chromosome and viruses (see references listed above). Moreover, the potentiality of using CXDM based on X-ray free-electron lasers for single biomolecule imaging has been well discussed recently in the literature as a potential tool for revealing these structures (Neutze *et al.*, 2000 ▶; Miao *et al.*, 2001 ▶; Fung *et al.*, 2008 ▶). Here we provide a detailed account of the instrumentation, the experimental procedures and the post-experimental data analysis in CXDM that yield high-quality image reconstructions.

## 2. Experimental set-up

Synchrotron facilities offer a few advantages over other X-ray sources, *i.e.* small beam divergence, continuous energy modulation and high beam flux, and thus greatly facilitate the acquisition of high-resolution data. Such beam characteristics are due to the underlying sophisticated ring and beamline design. For providing an overall view of the instrument, we first describe the overall structure of the coherent X-ray optics (BL29XUL) beamline (Tamasaku *et al.*, 2001 ▶) at SPring-8, and then detail the corresponding CXDM set-up. BL29XUL has three major parts: front-end, optics hutch and three experimental hutches (EH1, EH2 and EH3). At the front-end a standard in-vacuum undulator insertion device is located, with tunable 140 period magnets resulting in an optimized X-ray flux emission (∼10^13^ photons s^−1^) and covering a spectral range of 4.5 to 18.7 keV. A liquid-nitrogen-cooled Si double-crystal monochromator and a pair of reflecting mirrors occupy the optics hutch and are used to control the spectral-flux modulation and beam collimation.

**Figure 1:**
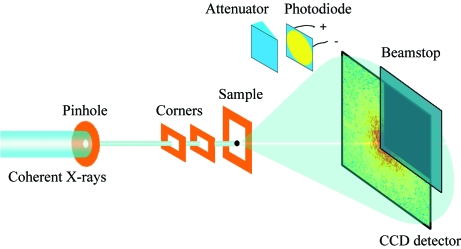
Schematic layout of the X-ray diffraction microscope. A 20 µm pinhole and two guard corners are used to define a clean X-ray beam. The oversampled diffraction pattern is measured by a CCD camera with 1340 × 1300 pixels and a pixel size of 20 µm × 20 µm. A beamstop is used to block the direct beam for protecting the detector and to avoid pixel saturation near the beamstop which would result in severely smeared patterns. An attenuator and a photodiode are used to align the microscope to the X-rays.

The experimental hutch (EH1) houses the customized diffraction microscope, which is located ∼52 m from the X-ray source. Figs. 1 ▶ and 2 ▶ show the schematic layout and photographs of the diffraction microscope instrument. The incident beam delivered to EH1 is 0.7 mm (V) × 1.3 mm (H) in size which is a relatively large area when the typical sample size of ∼1–10 µm is considered. To enhance spatial coherence, a 20 µm-diameter pinhole aperture is installed at ∼1 m upstream of the sample (Kohmura *et al.*, 2005 ▶). Downstream of the pinhole, two thick silicon windows with bevelled edges are introduced inside the sample chamber as L-shaped guard corners, where the lower-right-hand corners are used to minimize the scattering from the pinhole edges. The combination of pinhole and corners produces a clean diffraction signal in three adjacent quadrants on the detector. A movable attenuator, positioned downstream of the sample, permits a direct beam-position measurement, and also allows the alignment of other optical components. The location of the incident beam facilitates the estimation of the central pixel of measured diffraction patterns, which would be further refined at the data analysis procedure discussed in the following section. At the downstream end of the attenuator a movable photodiode is positioned to allow alignment of the optical components of the microscope. The diffraction patterns are measured by a deep-depletion and liquid-nitrogen-cooled CCD camera with 1340 × 1300 pixels and a pixel size of 20 µm × 20 µm (PI-LCX1300). The distance between the sample and the CCD camera is adjustable in order to fulfil the oversampling requirement (Miao *et al.*, 1998 ▶), which is a function of the X-ray wavelength, the sample size and the detector pixel size (Miao *et al.*, 2003*b* ▶). A large beamstop, mounted just in front of the CCD detector, is used to block the fourth noisy quadrant as well as the direct beam. To minimize the beam attenuation owing to air molecules and to reduce the background signal, the CXDM is operated in a vacuum (∼10^−4^ Pa). In-vacuum piezo-actuator coupled motion stages (Newport CMA-25) with a resolution of 1 µm per step are used to manipulate the guard corners and sample positions. Motion stages are controlled by a program written using commercial software (*LabView*).

The sample is mounted on a thin (30 nm) silicon nitride membrane framed by 200 µm-thick Si. The thin membrane is a good sample support because of its low absorption at X-ray energies. One imperative requirement for CXDM is that the sample be well isolated on the membrane and that the membrane be free from films, residue, dust and condensate. Non-uniformities upon the membrane near the sample will result in interference measured in the diffraction pattern that may hinder the reconstruction. Prior to mounting the sample inside the chamber, its relative position referenced by the membrane edges is mapped using an optical microscope.

As the membrane edges are the only landmark for the sample location, scanning the sample stage to find the designated edges is subsequently performed. This step is performed using the photodiode as the intensity counter. Once the edge position is known, the sample can be located using the offset coordinates obtained from the optical microscope mapping. Fine adjustment of the sample position is carried out later by maximizing the counts recorded on a CCD detector. A two-dimensional scanning of the sample is performed in the plane perpendicular to the beam direction. A low-resolution diffraction pattern is measured by the CCD for each scanning position of the sample. The best sample position is located, corresponding to the maximum diffraction intensity. In order to measure a three-dimensional data set, the sample stage is mounted on a rotary stage. Owing to interference of the edges of the silicon-nitride membrane support with the X-ray beams, the sample is usually rotated between ±70° and ±80° in actual experiments.

## 3. Data acquisition

**Figure 3:**
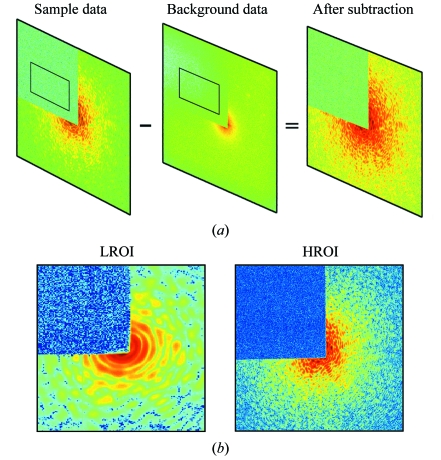
(*a*) A clean diffraction pattern (right) is obtained by subtracting the background pattern (middle) from the raw diffraction pattern (left). The rectangular regions are used to determine the scaling factor. (*b*) LROI (left) and HROI (right) diffraction patterns are measured to enhance the dynamic range of the diffraction intensity. The size of the LROI pattern is typically around 200 × 200 pixels, and the acquisition time is ∼0.5 s per exposure with a few thousand exposures. The short exposure time is to reduce the missing intensity at the centre of the diffraction pattern which is critical in phase retrieval (Miao *et al.*, 2005 ▶). To measure the HROI pattern (1340 × 1300 pixels) the beamstop is further moved in to block the low-resolution intensity, allowing us to increase the exposure time up to minutes. To enhance the high-resolution signal, we usually accumulate tens of exposures to obtain a final HROI pattern.

Data acquisition is divided into two key stages: low-resolution and high-resolution acquisition. Owing to a large dynamic range of the diffraction intensity across the frequency spectrum but a limited dynamic range which can be covered by present CCD detectors, it is best to measure these regions separately as shown in Fig. 3(*b*) ▶. The high-resolution data are more readily registered by the CCD when the beamstop completely blocks the inner speckles of the pattern. We term this data HROI (high-*Q* region of interest), with typically *Q* ≥ 0.03 nm^−1^, where *Q* = 4πsin(θ)/λ and 2θ is the diffraction angle. Similarly, we denote the complementary data LROI (low-*Q* region of interest), with typically *Q* ≤ 0.04 nm^−1^. Note that the *Q*-range for HROI and LRIO is sample dependent. The overlapping *Q*-range between HROI and LROI is used to align and normalize the two patterns. Backgrounds for LROI and HROI are acquired separately and immediately after the corresponding data are obtained. That is, we first measure the LROI data, shift the membrane so that the beam passes through a blank region of the membrane, and record the LROI background. We then revert to the sample position (which may have to be re-optimized), measure the HROI data, and record the HROI background. As demonstrated before, when the missing intensity data is confined within the centro-speckle, a specimen’s image can be reliably reconstructed from the diffraction pattern (Miao *et al.*, 2005 ▶). Thus the LROI data should be acquired first since optimizing the missing centre data will determine the viability of the diffraction pattern. The optimization consists of modifying the guard corners and beamstop positions such that a clean beam interacts with the sample to produce a clean speckle pattern as well as a small missing centre. Fig. 3(*b*) ▶ shows two exemplary LROI and HROI diffraction patterns. To obtain a three-dimensional data set the sample is rotated around a single tilt axis. At each tilt angle the sample is aligned to the incident beam, and the same data acquisition procedure is repeated.

## 4. Data analysis

A true test of a successful experiment lies in the quality of a reconstructed image, which can be quantified in terms of a Fourier space *R*-factor of the reconstruction and the correlation between independent reconstructions from initial random phase sets. The *R*-factor (*R*_f_) is calculated for the reconstructed image, defined as
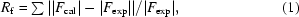
where |*F*_cal_| and |*F*_exp_| represent the calculated and experimental Fourier modulus, respectively. However, obtaining a final image requires analyzing and processing the acquired measurements. Although no amount of data analysis can correct poor measurements, high-quality data may yield undesirable reconstructions if not analyzed well. Hence these pre-reconstruction procedures play an important role in CXDM, and here we detail their consecutive steps.

### 4.1. Background subtraction

In order to take care of stray scattering, CCD thermal and read-out noises and non-uniformities across a CCD chip, we start by subtracting the background. This step is performed independently for HROI and LROI data. When the background exposure is shorter than the measurement exposure, we rescale accordingly. Although one may use various approaches to data normalization, we have found the following approach to be the most robust. Let us call the measured intensities *I*_M_(*k*_*x*_, *k*_*y*_), the background *I*_B_(*k*_*x*_, *k*_*y*_) and the rescaling factor α, which can be found by minimizing the difference between *I*_M_(*k*_*x*_, *k*_*y*_) and *I*_B_(*k*_*x*_, *k*_*y*_) over a rectangular region *S* located behind the beam stop shown in Fig. 3(*a*) ▶. Using the least-square minimization
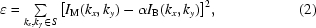
we calculate its derivative and set it equal to zero which determines the scale factor α,
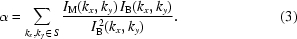
Because the data behind the beamstop are significantly attenuated and do not have characteristic diffraction features, the corresponding background and signal measurements are roughly proportional to the incident beam and the exposure time. Under ideal conditions *I*_M_(*k*_*x*_, *k*_*y*_) and *I*_B_(*k*_*x*_, *k*_*y*_) behind the beamstop are identical after rescaling. Then we normalize the intensities to exposure time Δτ,
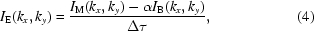
where *I*_E_(*k*_*x*_, *k*_*y*_) is the normalized experimental diffraction pattern after background subtraction.

### 4.2. Merging the LROI and the HROI data

As we have optimized the dynamic range of the detector by collecting data in two different regions, we face the delicate task of seamlessly merging these two patterns. Hence, we start by roughly aligning the two sets, using the direct beam position as the common centre. Then we select a boot-shaped region where the two sets overlap. Such a geometrical region allows for a large sampling of the overlapped high-signal area.

**Figure 4:**
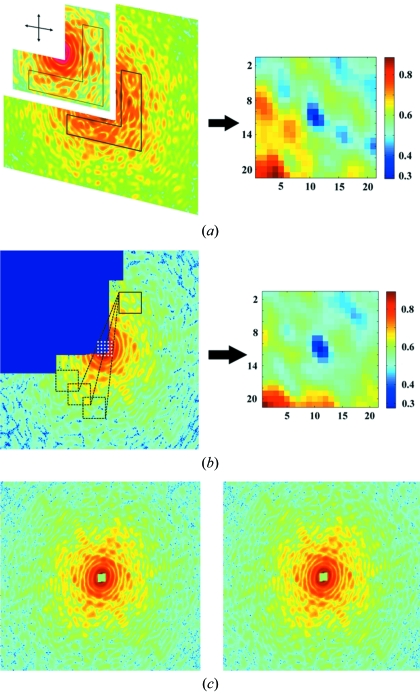
(*a*) Merging of the LROI and HROI diffraction patterns. An overlapping region (*i.e.* the boot-shaped region) is used to align the LROI and HROI patterns by minimizing the error distribution map (right). (*b*) Localization of the centre pixel of the diffraction pattern by using centrosymmetry. The error distribution map (right) indicates the optimal centre pixel. (*c*) A complete diffraction pattern after the missing quadrant is recovered by using centrosymmetry (left). The first and third quadrant data are averaged to enhance the signal-to-noise ratio of the diffraction pattern (right).

As the LROI exposure is shorter than the HROI exposure, we also rescale the LROI following the least-square minimization procedure as described in the previous section. To find the best data merging, we test all the possible alignments in the vicinity of the initial aligning as described above. Here, we deploy an integer shift method to enumerate all the possible alignments. Fig. 4(*a*) ▶ shows the fixed overlapped region and the corresponding error distribution map.

### 4.3. The missing quadrant

In our experimental set-up the intensity in the fourth quadrant is blocked by a beamstop, which can still be recovered by taking advantage of the centrosymmetry of a diffraction pattern. To recover the data in the fourth quadrant, we first locate the centre of the diffraction pattern. In ideal situations the beam position can be determined by taking a CCD image of the incident beam with the attenuator in. However, as the beam may drift with time, this value will not be satisfactory. Hence we rely on a measured diffraction pattern to accurately recover the centre.

For centrosymmetric data, the centre may be found by inducing this symmetry as follows. We choose a region in the vicinity of the centre, and compare it with possible match regions to find the best match. Fig. 4(*b*) ▶ shows the fixed region to the top right and the compared region to the bottom left and the error distribution map. Once the centre is determined, the missing quadrant data can be recovered by rotating the respective fourth quadrant [Fig. 4(*c*) ▶, left]. For the first and third quadrant data we average them to enhance the signal-to-noise ratio. As Fig. 4(*c*) ▶ (right) shows, in practice we again verify the quality of the pre-determined centre as well as the centrosymmetry of data itself before averaging the signal.

### 4.4. Data binning and deconvolution

For largely oversampled data we can bin the pattern in order to enhance the signal-to-noise ratio, yet yielding an exact pattern by numerical deconvolution (Song *et al.*, 2007 ▶). The binning operation means taking *m* × *n* pixels and averaging their values in a new pixel. To preserve the centre pixel and hence avoiding the use of fractional Fourier shift, we recommend *m* and *n* to be odd numbers. Before binning, the missing centre has to be filled in by using iterative algorithms. After the binning we perform deconvolution to the assembled diffraction pattern to remove the effect of the finite size of the CCD pixel (Song *et al.*, 2007 ▶). Finally, we set the missing central data to unknown pixels, set negative values to zero and convert the experimentally measured intensity into the Fourier modulus, which is used for phase retrieval to regain both missing data and the phase information.

## 5. Results

**Figure 5:**
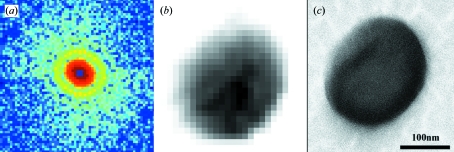
(*a*) X-ray diffraction pattern obtained from a single unstained herpesvirus virion. The diffraction pattern, displayed on a logarithmic scale, extends to *Q* = 0.28 nm^−1^ at the edges. (*b*) The corresponding image reconstructed from (*a*). (*c*) SEM image of the same virion, where (*b*) and (*c*) are displayed on the same scale.

**Figure 6:**
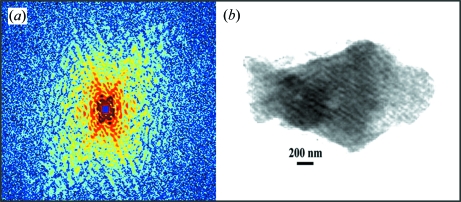
(*a*) X-ray diffraction pattern of a highly mineralized fish bone particle. The diffraction pattern, displayed on a logarithmic scale, extends to *Q* = 0.26 nm^−1^ at the edges. (*b*) The reconstructed image from (*a*) where the striations represent the mineralized fibrils.

By using the X-ray diffraction microscope mounted on BL29XUL at SPring-8, we have measured oversampled diffraction patterns from various materials science and biological samples. Here we illustrate two representative examples: coherent X-ray diffractive imaging of herpesvirus virions (Song *et al.*, 2008 ▶) and intramuscular fish bone (Jiang *et al.*, 2008 ▶). Following the experimental procedures and data analysis described above, we obtained two post-analysis diffraction patterns from a single unstained herpesvirus virion (Fig. 5*a* ▶) and a highly mineralized bone particle (Fig. 6*a* ▶). The diffraction patterns, displayed on a logarithmic scale, extend to *Q* = 0.28 nm^−1^ and *Q* = 0.26 nm^−1^, respectively, at the edges. To enable direct phase retrieval, we ensured the missing centres are confined within the centro-speckle of the diffraction patterns.

The phase retrieval of the diffraction pattern was conducted using the guided hybrid input and output algorithm (GHIO) (Chen *et al.*, 2007 ▶). The GHIO algorithm began with 16 independent reconstructions with random phases as the initial input. After ∼2000 iterations, 16 images (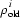, *i* = 1, 2,…, 16) were reconstructed, which were defined as the zeroth generation. The image with the smallest *R*_f_ was chosen as a seed (ρ_seed_). Sixteen new images (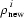) were obtained using 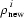 = 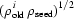. The 16 new images were used as the initial input for the next generation. Usually after nine generations the 16 reconstructed images became consistent, and the best five images with the smallest *R*_f_ were averaged to be the final image. Fig. 5(*b*) ▶ shows the final reconstruction of a single unstained herpesvirus virion. Compared with the scanning electron micrograph (SEM) image (Fig. 5*c* ▶), the reconstructed image exhibits a lower spatial resolution (∼22 nm), but shows the internal structure [the dark area near the centre in Fig. 5(*b*) ▶], which is likely to be the capsid of the herpesvirus virion. Furthermore, by measuring the incident and diffracted X-ray flux, the quantitative electron density map of the virion can be directly calculated from the reconstructed image (Song *et al.*, 2008 ▶). To further improve the resolution, more intense coherent X-rays and cryogenic technologies (Huang *et al.*, 2009 ▶; Lima *et al.*, 2009 ▶) are needed in order to measure the high-resolution diffraction intensity while reducing radiation damage effects to the sample. Fig. 6(*b*) ▶ shows the reconstructed image of a highly mineralized bone particle with a resolution of 24 nm. The striations in the figure represent the mineralized fibrils, which are almost parallel to each other. Since the bone particle is at the late stage of mineralization, the mineral crystals fill up the space between the collagen molecules and form fully calcified collagen fibrils (Jiang *et al.*, 2008 ▶).

## 6. Conclusion

Using coherent X-rays from the BL29XUL undulator beamline at SPring-8 and a specially designed coherent diffraction microscope, we have obtained high-quality diffraction patterns from various materials science and biological samples. The experimental set-up and the data analysis procedures associated with this coherent diffraction microscope, and the subsequent structure reconstructions, have been presented. While we focus on the applications of CXDM with synchrotron radiation, the instrumentation and the data analysis procedures described here are in principle applicable to other coherent X-ray sources such as table-top high-harmonic generation, soft X-ray lasers and X-ray free-electron lasers.

## Figures and Tables

**Figure 2:**
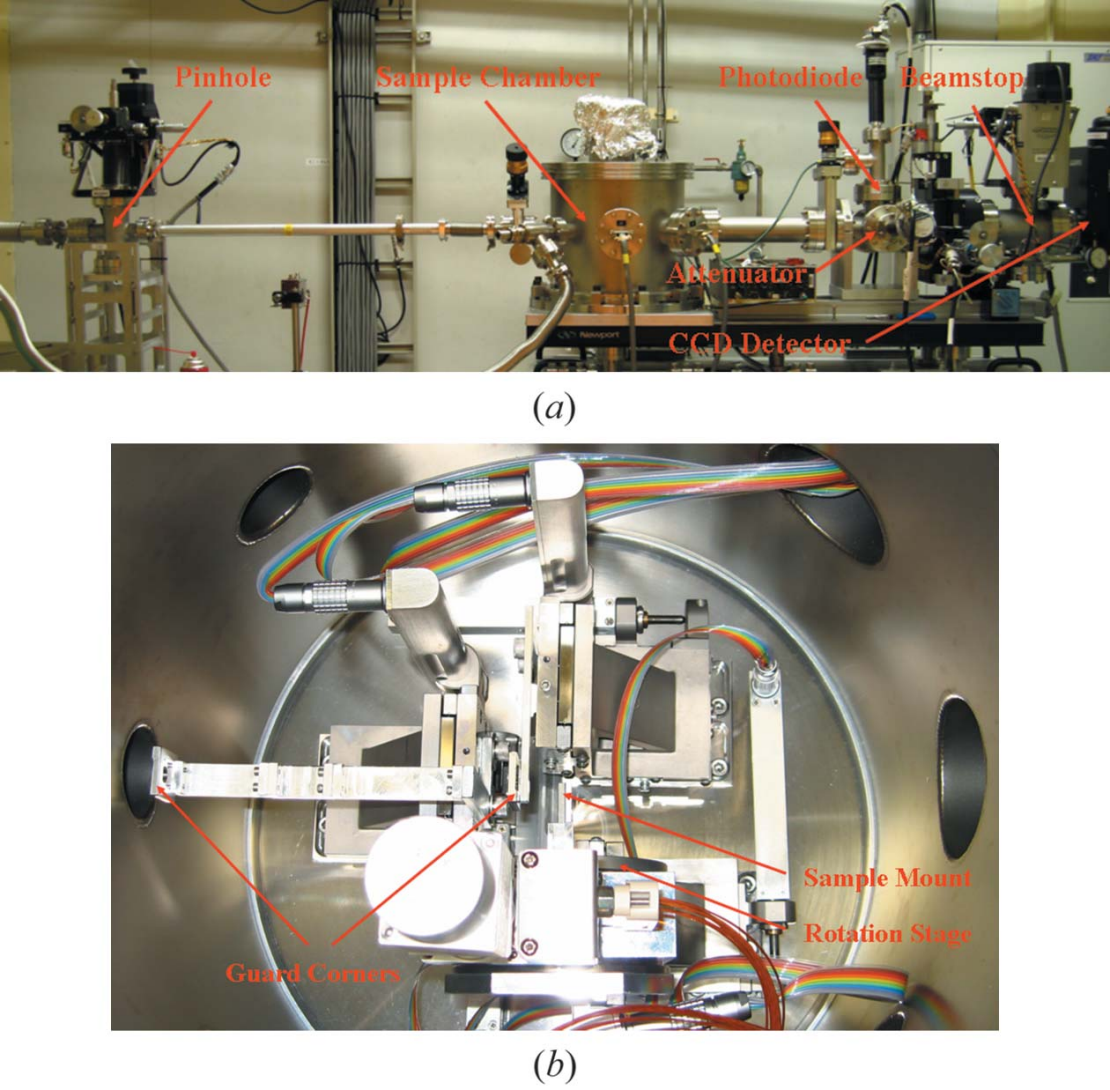
(*a*) Photograph of the X-ray diffraction microscope mounted on an undulator beamline (BL29XUL) at SPring-8, including a pinhole, a sample chamber, a photodiode, an attenuator, a beamstop and a CCD camera. (*b*) Photograph of the sample chamber, including two guard corners, a sample mount and a rotary stage.
